# Discovery and Evolutionary Analysis of a Novel Bat-Borne Paramyxovirus

**DOI:** 10.3390/v14020288

**Published:** 2022-01-29

**Authors:** Wentao Zhu, Yuyuan Huang, Xiaojie Yu, Haiyun Chen, Dandan Li, Libo Zhou, Qianni Huang, Liyun Liu, Jing Yang, Shan Lu

**Affiliations:** 1 State Key Laboratory of Infectious Disease Prevention and Control, National Institute for Communicable Disease Control and Prevention, Chinese Center for Disease Control and Prevention, Beijing 102206, China; wentaozhu@126.com (W.Z.); hyylhm@163.com (Y.H.); Huangqiannini@163.com (Q.H.); liuliyun@icdc.cn (L.L.); 2 Hainan Provincial Center for Disease Control and Prevention, Haikou 570203, China; yxjie2008@sina.com (X.Y.); Chenhaiyun1682008@163.com (H.C.); HNCDClidandan22@126.com (D.L.); sdzqbotao@163.com (L.Z.); 3 Shanghai Public Health Clinical Center, Fudan University, Shanghai 201508, China; 4 Research Units of Discovery of Unknown Bacteria and Function, Chinese Academy of Medical Sciences, Beijing 100730, China

**Keywords:** paramyxovirus, *Jeilongvirus*, bat, co-evolutionary, virus discovery

## Abstract

Paramyxoviruses are a group of RNA viruses, such as mumps virus, measles virus, Nipah virus, Hendra virus, Newcastle disease virus, and parainfluenza virus, usually transmitted by airborne droplets that are predominantly responsible for acute respiratory diseases. In this paper, we identified a novel paramyxovirus belonging to genus *Jeilongvirus* infecting 4/112 (3.6%) bats from two trapping sites of Hainan Province of China. In these animals, the viral RNA was detected exclusively in kidney tissues. This is the first full-length *Jeilongvirus* genome (18,095 nucleotides) from bats of genus *Hipposideros*, which exhibits a canonical genome organization and encodes SH and TM proteins. Results, based on phylogenic analysis and genetic distances, indicate that the novel paramyxovirus formed an independent lineage belonging to genus *Jeilongvirus*, representing, thus, a novel species. In addition, the virus-host macro-evolutionary analysis revealed that host-switching was not only a common co-phylogenetic event, but also a potential mechanism by which rats are infected by bat-origin *Jeilongvirus* through cross-species virus transmission, indicating a bat origin of the genus *Jeilongvirus*. Overall, our study broadens the viral diversity, geographical distribution, host range, and evolution of genus *Jeilongvirus*.

## 1. Introduction

Most orthoparamyxoviruses are known to cause diseases in their respective hosts with several of them known as zoonotic pathogens [1]. Emerging outbreaks of several zoonotic paramyxoviruses, such as measles virus, Nipah virus, mumps virus, and Hendra virus, have caused severe diseases and high fatality rates in humans [2]. Additionally, it is important to notice that the *Paramyxoviridae* is a family of negative-sense, non-segmented, large-enveloped RNA viruses consisting of 17 genera within four subfamilies [3,4,5]. The paramyxoviruses genomes range from 14,296 to 20,148 nucleotides (nt) in length, which encode a gene arrangement (3′-to-5′) of 6–10 proteins, including six structural proteins: nucleocapsid (N), phosphoprotein (P), matrix protein (M), fusion protein (F), glycoprotein (G), RNA polymerase (L), and three accessory proteins, such as: C protein (C), small hydrophobic protein (SH), and transmembrane protein in some members [3,6,7]. The genus *Jeilongvirus*, belonging to the subfamily *Orthoparamyxovirinae* within family *Paramyxoviridae*, is a newly established genus in 2020, and it currently includes seven species with accessory SH and/or TM proteins between F and G proteins [3,8].

Paramyxoviruses have been detected mainly in vertebrates (mostly in mammals and birds), and examples have also been identified in reptiles and fishes [3,4]. The majority of the bat paramyxovirus species are identified in bats within the suborder *Megachiroptera* (large Old World fruit bats) and in Eurasian bats [9,10,11]. Indeed, a variety of rats and bats are the ones that serve as the main reservoir hosts of genus *Jeilongvirus* to date [9,12]. The great roundleaf bat (*Hipposideros armiger* is a bat species within family *Hipposideridae* of suborder *Microchiroptera*, which is found in South Asia, Southeast Asia, and China. Until today, only one virus (BtHp-ParaV/GD2012, KJ641655) in genus *Jeilongvirus* was detected in bats of family *Hipposideridae* (*Hipposideros pomona*), for which only partial genome (4413 bp) has been characterized [13]. In addition to this, there are two viruses within genus *Jeilongvirus* detected in non-bat/rat hosts: Feline paramyxovirus from cats [14] and Belerina virus from hedgehogs [1], respectively.

This research investigates the presence and the evolutionary analysis of a novel paramyxovirus identified in bats (*Hipposideros armiger*) from Hainan Province of China, and has important implications for our knowledge of bat-borne paramyxovirus diversity.

## 2. Materials and Methods

### 2.1. Samples and Ethics

Between March and April 2021, 112 bats were collected from two abandoned caves in two sampling sites of Hainan. Bats were captured with nylon netting by humans wearing protective suits and put in airtight bags. Captured bats were anesthetized with ether and euthanized by cervical dislocation, then dissected in biosafety cabinets of the local Center for Disease Control and Prevention (CDC). Then, tissues (kidney, respiratory tract, brain, and intestinal tract) from these bats were collected aseptically, preserved in Hank’s balanced salt solution [15], and stored at −80 °C. The bat species were identified based on polymerase chain reaction (PCR) and Sanger sequencing, using primers targeting for NADH dehydrogenase subunit 1 (*ND1*) gene [16]. Sample collection and handling procedures were conducted as part of the local CDC surveillance programs and approved by the State Key Laboratory for Infectious Diseases Prevention and Control (ICDC-2021038).

### 2.2. RNA Extraction and Sequencing

A total of 30 mg of tissue samples were individually homogenized in phosphate buffer solution (PBS) using the TissueLyser II system (Qiagen, Hilden, Germany), and were centrifuged at 13,000× *g*, 4 °C for 15 min. The total RNA was extracted, according to manufacturer’s instructions, using RNeasy Plus Universal Mini Kit (Qiagen). RNA concentration of each sample was estimated using NanoDrop 2000 (Thermo, Waltham, MA, USA). The RNA of forty kidney samples were selected randomly and organized into one pool using same amount from each sample. The potential naked DNA was removed using DNase I (Life Technologies, Waltham, MA, USA) and, subsequently, rRNA was removed by the Ribo-Zero Gold rRNA Removal Kit. Libraries were constructed as previously reported [17], using the TruSeq Stranded Total RNA Library Prep Gold kit, following the manufacturer’s instructions. The high-throughput sequencing was conducted using the Illumina HiSeq2000 platform, yielding paired-end 150-bp (2 × 150 bp) reads. The resulting raw data were trimmed using Trimmomatic v0.32 [18] in order to remove reads with adapter sequences and low-qualified reads.

### 2.3. Viral Genome Characterization

The obtained clean data were de novo assembled using Trinity [19] and contigs were annotated by BLASTx against the virus database (taxid: 10239) using Diamond with e-value cut-off of 1 × 10^−5^ [20]. The open reading frames (ORFs) of near-complete genome were predicted by ORFfinder (https://www.ncbi.nlm.nih.gov/orffinder/, accessed on: 21 December 2021) and comparison with related paramyxoviruses using the online conserved domain database (https://www.ncbi.nlm.nih.gov/cdd, accessed on: 18 December 2021). The resulting nucleotide and amino acid sequences of putative viral proteins were confirmed by BLAST against the non-redundant protein database downloaded from NCBI (https://ftp.ncbi.nlm.nih.gov/, accessed on: 12 November 2021). The clean reads were mapped backed to the obtained genome to estimate the sequencing depth and coverage. While protein domains were searched by TMHMM [21] and InterPro [22], the glycosylation sites were predicted by a web server (https://services.healthtech.dtu.dk/, accessed on: 12 December 2021). After removing all the ambiguous region, the amino acid pairwise distance (p-distance) values of L proteins were estimated using MEGA v7 with variance estimated by bootstrap replications of 1000 [23].

### 2.4. PCR Screening and Confirmation of the Genome Sequence

Based on the viral contig sequences, paired primers (ParaFX: 5′-CAGGCAAAGCAGAGTTCAGC-3′, and ParaRX: 5′-CCATTGGATTTTCTTTACACTTC-3′) were designed to screen the novel paramyxovirus in samples. Reverse transcription polymerase chain reactions (RT-PCR) were conducted by OneStep RT-PCR Kit (Takara) and the PCR products were subjected to Sanger DNA sequencing. In addition to these methods, four paired primers (Table S1) targeting several regions of the genome were used to confirm the genome sequence.

### 2.5. Phylogenetic Analysis

The amino acid sequences of L, N, and P proteins of virus from this study and members of subfamily *Orthoparamyxovirinae* or genus *Jeilongvirus* were aligned using MAFFT program v7 [24]. Substitution models were assessed by ModelFinder according to Akaike information criterion (AIC) [25,26]. Thereafter, phylogenetic trees were reconstructed using PhyML 3.0 with Dayhoff model, based on maximum likelihood algorithm and bootstrap values of 1000 [27]. The resulting trees were visualized and modified in the Interactive Tree of Life (iTOL) (http://itol.embl.de/personal_page.cgi, accessed on: 21 December 2021).

### 2.6. Co-Divergence Analysis

In order to estimate the relative frequencies of co-divergence and cross-species transmission in the evolutionary history of genus *Jeilongvirus* with their hosts, we conducted co-phylogenetic analysis using Jane package v4 [28]. The phylogenetic tree of viruses was constructed based on L proteins as described above, while a tree of the host species was built using TimeTree [29]. As previously reported [30], we carried out two sets of the ‘event costs’ with one being 0 for co-divergence, 1 for duplications, 1 for host-switching, and 1 for extinction, and the other being 1 for duplication, 2 for host-switching, 1 for loss and 1 for failure to diverge.

## 3. Results

### 3.1. Discovery of a Novel Paramyxovirus

**Figure 1:**
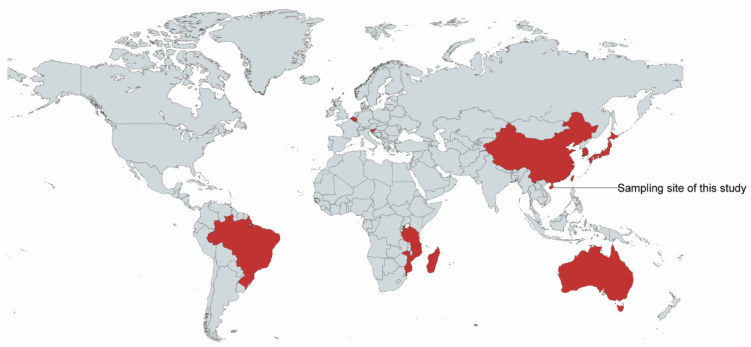
The distribution map of *Jeilongvirus* and the sampling sites of this study. The distribution regions of *Jeilongvirus* are labeled in red, while the sampling sites are marked with words.

RNA samples extracted from forty (40//112) kidney tissues of bats from the Hainan Province of China (Figure 1) were selected randomly, pooled in a same amount, and subjected to high-throughput sequencing. The pooled library resulted in 139,838,946 paired-end reads comprising 21.0 Gb clean data (Q30 ≥ 91.4%). A total of 373,440 assembled contigs were obtained. BLAST results indicated that five contigs (6597, 4035, 435, 354 and 207 bp, respectively) belonged to paramyxovirus, thus, we designed primers based on these contigs to screen this virus in each sample, which revealed that the viral RNA was detected in four (4/112, 3.6%) samples and the sequencing results from the four samples shared 100% similarity. However, the viral RNA was not detected in other tissue types, including respiratory tract, brain, and intestinal tract. To obtain the complete genome, we selected one sample to conduct high-throughput sequencing again, which produced 70,590,294 raw paired-end reads with 10.6 Gb clean data after trimming. The host species of the four paramyxovirus-positive samples was found to be the great roundleaf bat, also known as the great Himalayan leaf-nosed bat (*Hipposideros armiger*).

### 3.2. Genomic Characteristics

**Figure 2:**
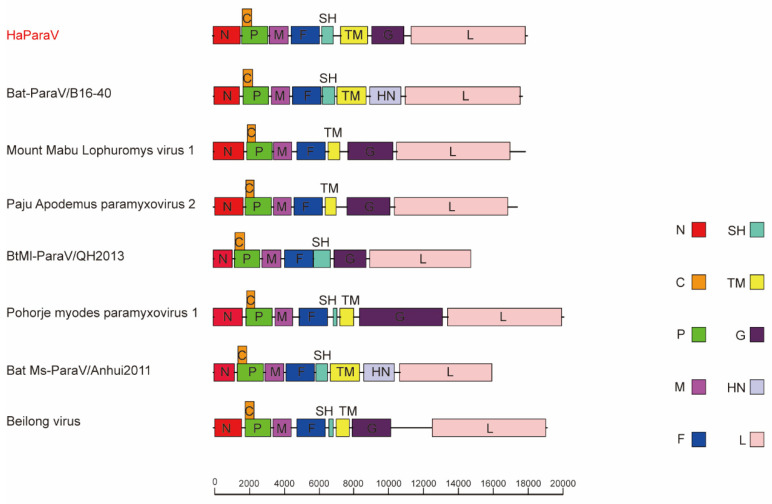
Comparisons between the genome organization of HaParaV and those of representatives of genus *Jeilongvirus*. The genomes are drawn with a unified length scale. The function of each protein is shown with a short description. The “HN” represents haemagglutinin-neuraminidase (HN) protein. The virus identified in this study is labeled with a red font.

The near-complete genome (accession number: OL630969) was 18,095-nt-long with a depth coverage of 488×, and named as Hipposideros armiger paramyxovirus (HaParaV). These high-throughput sequencing results were also confirmed by RT-PCR and Sanger sequencing. In some members of *Jeilongvirus*, the genomes are interspersed with one or two additional transcription units (encoding SH and/or TM proteins) between the F and G proteins. For HaParaV, the genomic sequence encoded nine ORFs (Figure 2), including those for both SH and TM proteins, a result that was identical to that observed in the closest relative of Bat paramyxovirus isolate Bat-ParaV/B16-40 (MG230624). However, the genome organization of HaParaV was different from the ones of several members of genus *Jeilongvirus* in lacking SH and/or TM proteins, as well as the length of G proteins (Figure 2). The G protein of HaParaV was homologous to members of haemagglutinin-neuraminidase superfamilies, which is a multi-functional protein that has receptor-binding, receptor-destroying, and membrane fusion activities, among others. Predicting results showed that the G protein of HaParaV contained six N-glycosylation sites in positions 12, 43, 128, 152 331, and 432, and 9 O-GalNAc glycosylation sites in positions 580–596 and 602. In addition, HaParaV had a unique sequence (NRKSCT) at the start of propeller blade 2, which was different from those of other members of *Jeilongvirus* (NRRSCT, NRRSCS or NRKSCS).

The pairwise amino acid comparisons between HaParaV with the corresponding homologous region of members within genus *Jeilongvirus* were performed and the results are shown in Table 1. The results reveal that the highest similarities were observed in M proteins (ranging from 58.4% to 77.1%) between HaParaV and related members (Table 1). However, HaParaV shared low homologies (<40% aa similarity) with members within genus *Jeilongvirus* in P, C, SH, and TM proteins.

### 3.3. Phylogenetic Analysis

**Figure 3:**
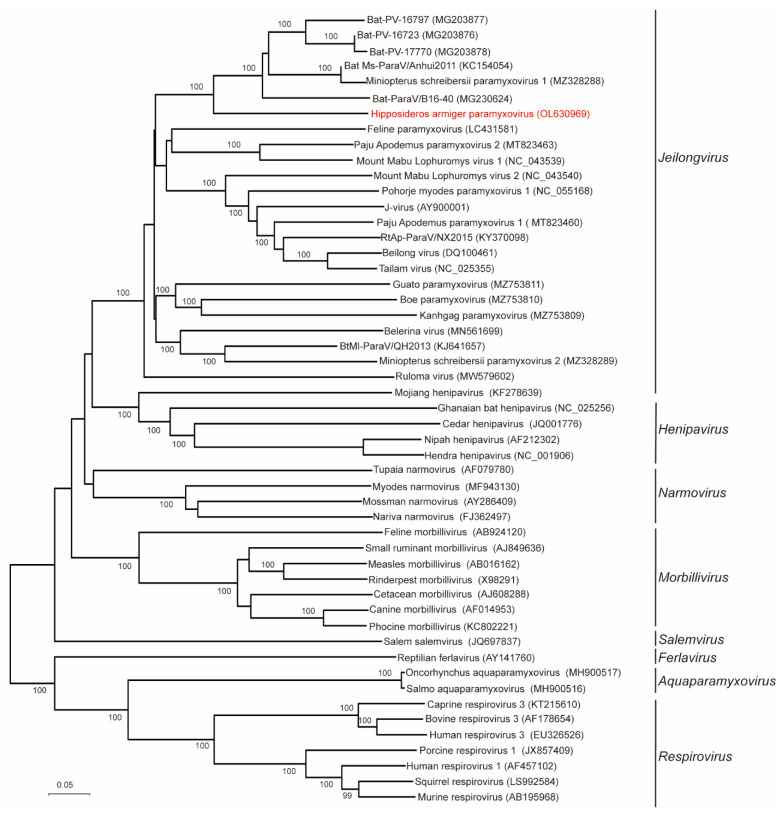
Phylogenetic analysis based on L proteins of subfamily *Orthoparamyxovirinae*. The evolutionary tree was inferred using amino acid sequences by the Maximum Likelihood algorithm with 1000 bootstraps. Only bootstrap values above 90% are shown. The accession numbers of each virus are labeled in brackets after corresponding viral names. The virus identified in this study is labeled with red font.

**Figure 4:**
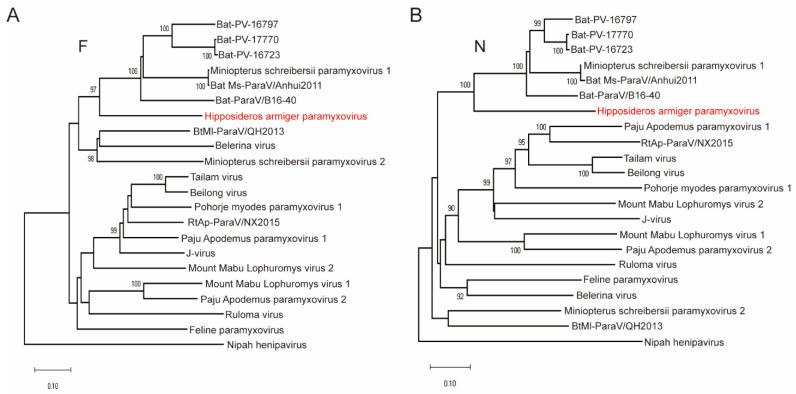
Phylogenetic analyses based on F (**A**) and N (**B**) protein amino acid sequences of genus *Jeilongvirus*, respectively. The evolutionary tree was inferred using amino acid sequences by the Maximum Likelihood algorithm with 1000 bootstraps. Only bootstrap values above 90% are shown.

The phylogenetic analyses were conducted based on the deduced amino acid sequences of L, F and N (Figure 3 and Figure 4) proteins, respectively. The resulting phylogenetic tree established based on L proteins of all members within subfamily *Orthoparamyxovirinae* revealed that HaParaV formed an independent clade within genus *Jeilongvirus*, and was closely related to Bat-ParaV/B16-40 (Figure 2). Additionally, the phylogenetic trees established based on F and N proteins showed similar results, indicating that HaParaV was a unique lineage and clustered with Bat-ParaV/B16-40 (Figure 3) within genus *Jeilongvirus*.

**Figure 5:**
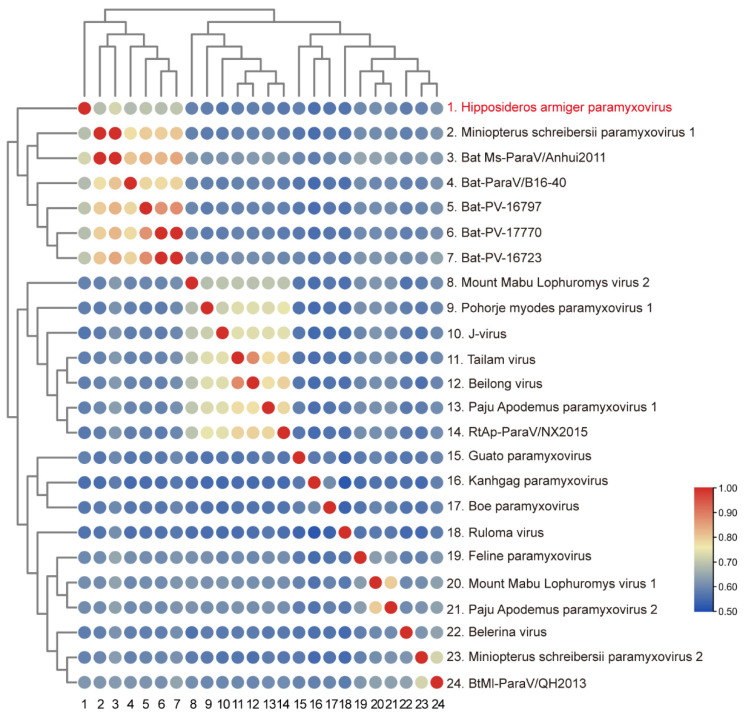
The genetic distances between the virus identified in this study and members of genus *Jeilongvirus*. The p-distance values are estimated using amino acid sequences of complete L proteins. The numbers (1–24) below the picture represent the corresponding viral names on the right side of the picture. The p-distance values were showed as colored circles. The rows and columns were clustered based on paired values. The scale values range from 0.5 to 1. The virus identified in this study is labeled with red font.

In addition, the genetic divergence at the amino acid level was estimated using complete L protein sequences. Results indicate that the values of p-distances between HaParaV and representative members of genus *Jeilongvirus* ranged from 0.56 to 0.73 (Figure 5), which supported that HaParaV was a novel species within genus *Jeilongvirus* [3].

### 3.4. Virus-Host Macro-Evolution of Genus Jeilongvirus

**Figure 6:**
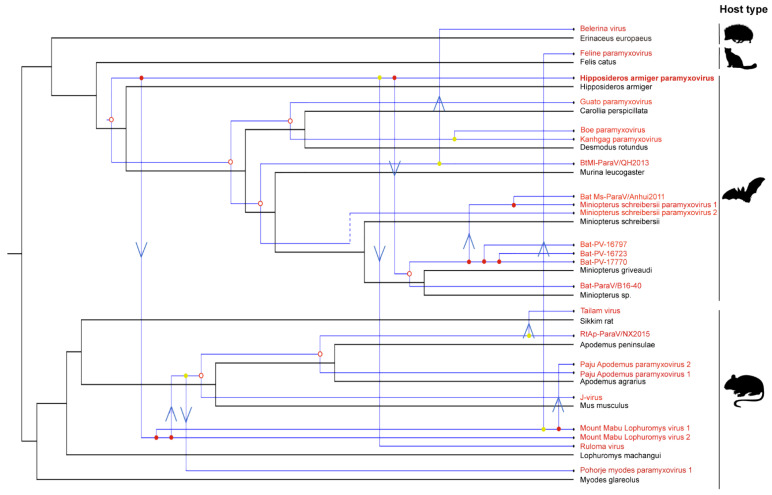
Host-virus co-phylogenetic analysis of *Jeilongvirus*. The virus identified in this study is labeled with red font. The host tree was inferred using TreeTime. The phylogenetic tree of viruses was built based on L proteins. Co-speciation, host-switching, duplication, and loss events are labeled with filled circles, arrows, empty circles, and dotted lines at the nodes, respectively. The colors of circles represent different placements: red for all other placements worse, and yellow for equally good placement exists. The names of the viruses are labeled with red font, and virus from this study is labeled with bold type. The small picture represents the corresponding host type.

In order to investigate the evolutionary history of genus *Jeilongvirus*, the tree topologies of viruses and their host species were compared (Figure 6). The event-based reconciliations revealed that the relative frequencies of the four tested evolutionary ‘events’ were 7 for co-divergence events, 10 for host-switching events, 5 for lineage duplications, and 1 for extinction, which are shown as a tanglegram between the virus and host phylogenies in Figure 6. Obviously, the majority of reconstructed events were host switching, which is not an unusual result of viral macro-evolutionary patterns as previously reported [31]. Moreover, the results show that the host species of genus *Jeilongvirus* mainly belonged to two clusters—bats and rats, which also indicated that viruses of bat origin infected rats through cross-species transmission in their evolutionary history (Figure 6). Furthermore, the obtained genome of HaParaV was the only full-length genome within genus *Jeilongvirus* detected from bats (*Hipposideros armiger*) of genus *Hipposideros* (Figure 6).

## 4. Discussion

The ongoing SARS-CoV-2 pandemic reminds us again about the powerful RNA viruses, which may spill over and adapt to human from animal reservoirs—especially bats [32]. Herein, based on high-throughput and Sanger sequencing, we have detected a novel bat-borne paramyxovirus belonging to genus *Jeilongvirus* from Hainan Province of China. Based on our knowledge, this is the first report of a full-length genome of *Jeilongvirus* from bats (*Hipposideros armiger*) within genus *Hipposideros* found in South Asia, Southeast Asia, and China. The phylogenetic analysis and p-distances (Figure 5) suggested that HaParaV formed a distinct branch, belonging to a novel species of genus *Jeilongvirus* [3]. The glycoprotein of *Jeilongvirus* is a multifunctional protein, including binging receptors, virus budding, and promoting fusion for F protein, and will be cleaved into cytoplasmic, transmembrane, stalk, and globular domains [33,34]. The propeller blade is the core region of globular domain of glycoprotein, which possesses activities of neuraminidase and binding receptor [35]. Thus, the unique start of propeller blade of HaParaV could have special influence on activity of glycoprotein. Previous experiments have demonstrated that Hendra and Nipah viruses are nephrotrophic in bats [36,37]. Indeed, this novel virus was detected exclusively in kidney tissues of bats, exhibiting an identical tissue distribution pattern to Kanhgág virus, Boe virus, and Guató virus within genus *Jeilongvirus*. However, further studies are necessary to prove that they are indeed nephritic tropism, and determine the potential pathogenicity of HaParaV along with other recently detected Jeilongviruses.

At long-term virus-host co-divergence, it is easier to investigate the evolutionary history of RNA viruses using single functional gene, such as RNA polymerase (*RdRp*), capsid, and glycoprotein, rather than whole viral genomes [38]. Moreover, phylogenetic trees are necessarily inferred using the relatively conserved gene, usually replicase sequences for RNA viruses [38,39,40]. In addition, although strict virus-host macro-evolution could not be always speculated and host switching events were relatively high in some cases, virus-host co-divergence over long evolutionary timescales was also probable [38]. Additionally, the evolutionary history of RNA viruses is commonly thought to be characterized by both host-switching and co-speciation [38,41]. Thus, this also seems to be true in paramyxoviruses. The results show that almost all host species of genus *Jeilongvirus* were divided into two clusters: bats and rats (Figure 6). All rodent-borne *Jeilongvirus* seems to have originated from an ancestral virus of bat-origin, which has the ability of interspecies transmission. We infer a hypothesis that HaParaV and rodent-borne jeilongviruses may have a common ancestor, or rodent-borne jeilongviruses may come from the lineage of HaParaV. This is evidenced by the evolutionary reconciliations, which strongly suggested a bat origin of the genus *Jeilongvirus*, highlighting the importance of bats as ancestral hosts of genus *Jeilongvirus* and the ability of genus *Jeilongvirus* to infect genetically divergent hosts. However, the presented data did not support significant co-speciation for the viruses of genus Jeilongvirus and host species. Additionally, the evolutionary history of the full-length genomes may be more complicated and not necessarily identical to that of L proteins, especially when all viruses have not yet been discovered nor gone extinct [38].

In conclusion, our study characterized a novel paramyxovirus from a new bat species from Hainan Province of China, and suggested the bat origin of genus *Jeilongvirus*, expanding the viral diversity, geographical distribution, host range, and evolution of genus *Jeilongvirus*. Additionally, this research underscored the importance of surveillance studies in order to prepare for the future emergence of yet unknown paramyxoviruses from wildlife reservoirs.

## Figures and Tables

**Table 1:** Similarities (%) at the amino acid sequence level between the novel paramyxovirus identified in this study and related paramyxoviruses within genus *Jeilongvirus*.

	N	P	C	F	M	SH	TM	G	L
Bat Ms-ParaV/Anhui2011	47.3	38.1	29.0	56.5	75.4	31.2	14.9	40.2	58.3
Bat-ParaV/B16-40	56.2	39.8	35.2	53.6	76.3	28.9	14.6	38.0	68.2
Bat-PV-16723	55.8	33.9	21.1	54.3	59.4	30.2	15.8	27.6	56.6
Bat-PV-16797	56.2	36.4	25.6	53.6	77.1	29.9	6.23	40.1	69.6
Bat-PV-17770	55.8	33.7	21.6	54.7	74.6	30.6	14.1	41.8	67.7
Beilong virus	38.2	27.2	20.1	47.4	60.3	4.2	4.3	25.0	57.3
Belerina virus	46.6	23.2	22.2	48.3	65.0	–	3.3	33.7	57.1
Boe paramyxovirus	44.1	27.7	24.4	50.0	69.8	–	–	31.2	56.2
BtMl-ParaV/QH2013	31.7	27.3	28.8	50.3	61.0	7.1	–	24.1	54.0
Feline paramyxovirus	30.0	29.6	26.7	48.5	67.0	–	3.7	20.5	59.3
Guato paramyxovirus	42.0	26.2	20.5	51.5	67.1	–	–	30.4	57.4
J-virus	35.8	24.9	20.5	47.1	59.5	1.9	5.6	27.7	56.1
Kanhgag paramyxovirus	44.2	25.6	27.3	49.7	66.4	–	–	33.0	55.5
Miniopterus schreibersii paramyxovirus 1	57.6	38.5	29.6	56.3	75.4	31.2	14.9	40.0	68.9
Miniopterus schreibersii paramyxovirus 2	45.7	–	–	50.9	62.3	8.5	–	29.0	58.1
Mount Mabu Lophuromys virus 1	38.9	31.2	25.6	49.5	63.7	–	3.7	22.4	60.0
Mount Mabu Lophuromys virus 2	39.7	23.4	22.7	48.2	62.6	–	5.1	26.2	58.6
Paju Apodemus paramyxovirus 1	39.3	25.6	21.6	49.7	61.2	3.2	4.3	11.9	57.6
Paju Apodemus paramyxovirus 2	42.5	30.9	29.6	47.2	66.3	–	3.1	21.9	59.8
Pohorje myodes paramyxovirus 1	35.7	26.1	19.9	49.0	58.4	3.7	3.5	12.5	57.9
RtAp-ParaV/NX2015	35.8	28.6	25.0	47.0	61.5	3.2	5.4	12.2	56.8
Ruloma virus	38.2	20.7	14.0	44.5	59.8	–	3.7	21.6	56.1
Tailam virus	38.7	27.6	19.9	49.2	60.6	4.6	3.5	18.2	57.5

## Data Availability

The resulting viral genome sequence from this study have been deposited in GenBank under accession number OL630969.

## Supplementary Materials

The following supporting information can be downloaded at: https://www.mdpi.com/article/10.3390/v14020288/s1, Table S1: Primers used in this study.
