# Supplementary material for: Discovery and Evolutionary Analysis of a Novel Bat-Borne Paramyxovirus
Source: Viruses. 2022 Jan 29;14(2):288. doi: 10.3390/v14020288 (PMC8879077; doi:10.3390/v14020288)
Supplement: Supplementary file 1 [file viruses-14-00288-s001.zip › viruses-1567746-supplementary.pdf]

Discovery and Evolutionary Analysis of a Novel Bat-borne Paramyxovirus

Wentao Zhu [1, #], Yuyuan Huang [1, #], Xiaojie Yu [2], Haiyun Chen [2], Dandan Li [2], Libo Zhou [2], Qianni Huang [1], Liyun Liu [1], Jing Yang [1, 3, 4 *], and Shan Lu [1, 3, 4 *]

[1] State Key Laboratory of Infectious Disease Prevention and Control, National Institute for Communicable Disease Control and Prevention, Chinese Center for Disease Control and Prevention, Beijing 102206, China

[2] Hainan Provincial Center for Disease Control and Prevention, Haikou 570203, Hainan, China

[3] Shanghai Public Health Clinical Center, Fudan University, Shanghai 201508, China

[4] Research Units of Discovery of Unknown Bacteria and Function, Chinese Academy of Medical Sciences, Beijing 100730, China

[#] These authors contributed equally to this work.

[*] Corresponding authors: lushan@icdc.cn (S. Lu) and yangjing@icdc.cn (J. Yang)

Supplementary Table S1 Primers used in this study.

Primer names	Length (bp)	Sequences
ParaF1	822	5′-TCCACTTGACAACATCGCCA-3′
ParaR1		5′-AGCAGAATGGTGGGCAATCA-3′
ParaF2	845	5′-TGCCATGATTGAGCCCTTGA-3′
ParaR2		5′-ATTCGGCAACTACCTGGCAA-3′
ParaF3	842	5′-GTCACAGCAGTGTTCCAACC-3′
ParaR3		5′-GCCACTTGGCAGCCTAAACT-3′
ParaF4	708	5′-GGTGTTGCATTGGGAGTTGC-3′
ParaR4		5′-TAGTTGTGTGCTGATGGGGG-3′
